# Redox-dependent control of i-Motif DNA structure using copper cations

**DOI:** 10.1093/nar/gky390

**Published:** 2018-05-24

**Authors:** Mahmoud AS Abdelhamid, László Fábián, Colin J MacDonald, Myles R Cheesman, Andrew J Gates, Zoë AE Waller

**Affiliations:** 1 School of Pharmacy, University of East Anglia, Norwich Research Park, Norwich NR4 7TJ, UK; 2 Centre for Molecular and Structural Biochemistry, University of East Anglia, Norwich Research Park, Norwich NR4 7TJ, UK; 3 School of Chemistry, University of East Anglia, Norwich Research Park, Norwich NR4 7TJ, UK; 4 School of Biological Sciences, University of East Anglia, Norwich Research Park, Norwich NR4 7TJ, UK

## Abstract

Previous computational studies have shown that Cu^+^ can act as a substitute for H^+^ to support formation of cytosine (C) dimers with similar conformation to the hemi-protonated base pair found in i-motif DNA. Through a range of biophysical methods, we provide experimental evidence to support the hypothesis that Cu^+^ can mediate C–C base pairing in i-motif DNA and preserve i-motif structure. These effects can be reversed using a metal chelator, or exposure to ambient oxygen in the air that drives oxidation of Cu^+^ to Cu^2+^, a comparatively weak ligand. Herein, we present a dynamic and redox-sensitive system for conformational control of an i-motif forming DNA sequence in response to copper cations.

## INTRODUCTION

**Figure 1.**
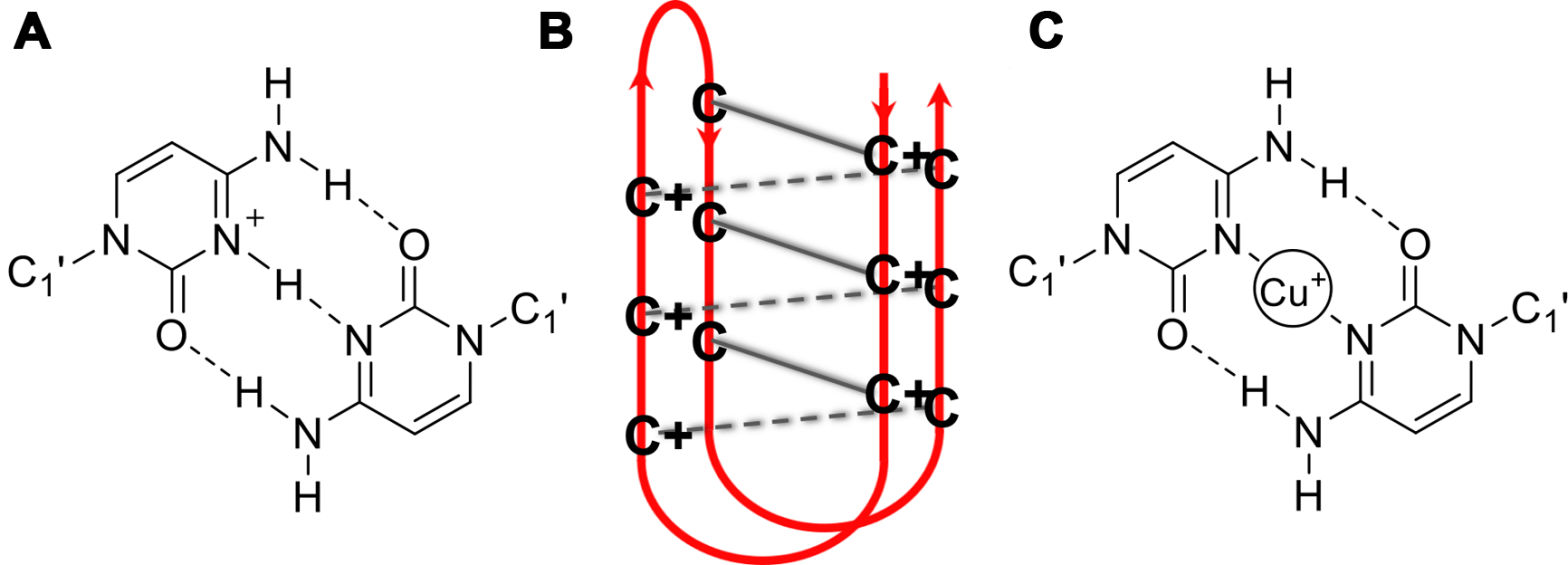
(**A**) Hemi-protonated C^+^-C base pair (**B**) i-motif structure (**C**) Proposed C–C base pair stabilized by Cu^+^.

Substantial interest and research has been devoted to studying the characteristics of the many non-canonical secondary structures that can be adopted by DNA. The ability of DNA to assume different conformations is controlled by the specific sequence of bases and the local environment (1). One such structure is the i-motif that forms from cytosine-rich DNA when two parallel duplexes containing cytosine repeats intercalate to form a quadruplex structure stabilized by C^+^-C base pairing (Figure 1) (2). These structures may readily form in acidic conditions where the N3 of cytosine can be protonated; subsequent intercalation and formation of the i-motif occurs rapidly (3). In 2003, this property was utilized to create the first proton-fuelled i-motif nanomotor (4). Since then, the i-motif has been exploited in the design of hundreds of pH-driven nanomachines (5,6) including an example of a light-driven pH-jump system (7) and a DNA nanomachine that can map spatial and temporal pH changes in living cells (8). There have been significant advances in the understanding i-motif structure and dynamics (9–11) and also the sequences (12–14) which can enable fine-tuning of the properties of these types of devices.

In addition to pH, Hg^2+^ (15) and Ag^+^ (16) have been used as alternative triggers for i-motif formation, but generally studies into the influence of cations on i-motifs are limited. It is known that alkali metals destabilize the structures (17,18) and our own work has revealed that Cu^2+^ is capable of re-folding i-motif forming sequences into a hairpin structure, even competing with acid-stabilized i-motif at low pH (19). Recently, Oomens and co-workers used infrared ion spectroscopy in combination with density functional theory (DFT) calculations to show that cytosine monomers in the presence of Cu^+^ form C–Cu^+^–C structures, analogous to the hemi-protonated C-dimers at the core of the i-motif (Figure 1C) (20). In contrast to alkali metal ions, that induce a different dimer conformation which sacrifices hydrogen-bonding interactions between bases for improved chelation of the metal cation, the C–Cu^+^–C dimer complex was proposed to be stable (20). Given the requirement for C–C base pairs in i-motif, building from this work we decided to investigate the effects of Cu^+^ on an i-motif forming DNA sequence.

## MATERIALS AND METHODS

### General experimental

Oligonucleotides hTeloC and hTeloC_FRET_ were purchased from Eurogentec and were HPLC purified. Solid DNA samples were initially dissolved as a stock solution in MilliQ water (100 μM for labeled and 1 mM for unlabeled); further dilutions were carried out in the respective sodium cacodylate buffer. Samples were thermally annealed in a heat block at 95°C for 5 min and left to cool slowly to room temperature overnight.

Experiments requiring anoxic conditions were performed under nitrogen in a UNIlab Plus glove box workstation with the oxygen level maintained below 0.5 ppm. Solutions of Cu^+^ were prepared in the glove box by dissolving solid CuCl in 0.1 M HCl and 1 M NaCl solution to double the desired concentration and then diluted to the final concentration using 2× sodium cacodylate buffer (final dilution using buffer prevents pH change on cation addition during experiments). Solutions of Cu^2+^, diethyldithiocarbamate (DETC) and sodium ascorbate were prepared by dissolving solid Cu_2_SO_4_, DETC or sodium ascorbate in MilliQ water, respectively.

### UV spectroscopy

UV spectroscopy experiments were performed on an Agilent Technologies Cary 4000 UV-Vis spectrophotometer and recorded using an open-top screw-cap 10 mm quartz cuvette with a silicone rubber septum to exclude air on transfer from the glove box to the spectrophotometer. Samples (1 ml) were diluted to 2.5 μM hTeloC in 10 mM sodium cacodylate buffer at the desired pH. Cu^+^ was added in 0.5 μl aliquots, and mixed, using a pipette to the desired concentration. Spectra were recorded over a wavelength range of 400–200 nm at room temperature in the absence of cation then after each addition, and zero corrected at 400 nm. The difference spectra at either pH were calculated by subtraction of the final folded spectrum, in the presence of Cu^+^, from the spectrum in the absence of Cu^+^.

### Circular dichroism

Circular dichroism (CD) spectra were recorded on a Jasco J-810 spectropolarimeter using a 1 mm path length quartz cuvette with a neck and a silicone stopper to exclude air on transfer from the glove box to the spectropolarimeter. hTeloC was diluted to 10 μM in 50 mM sodium cacodylate buffer at the desired pH to a total volume of 200 μl. The scans were performed at room temperature over a wavelength range of 200–320 nm with a scanning speed of 200 nm/min, response time of 1 s, 0.5 nm pitch and 2 nm bandwidth. A blank sample containing only buffer was treated in the same manner and subtracted from the collected data. Cu^+^ was added in 0.5 μl aliquots, and mixed, using a pipette to the desired concentration. For the chelator titration 150 μM Cu^+^ was added via a 1 μl addition from a 30 mM stock solution, DETC was then added in 0.5 μl aliquots to the desired concentration as above. Control spectra of DETC in buffer and DETC with hTeloC were also measured to confirm DETC itself had no effect on the spectra or the conformation of the DNA. The CD spectra represent an average of three scans and are zero corrected at 320 nm. For the redox experiments, each component was added via a 1 μl addition from a stock solution prepared at the concentration needed to yield the desired concentration. Control spectra of sodium ascorbate in buffer and sodium ascorbate with hTeloC were also measured to confirm that sodium ascorbate itself had no effect on the spectra or the conformation of the DNA. Titration experiments were performed at least in triplicate and processing of the data was carried out using Origin.

### FRET melting

The labeled oligonucleotide hTeloC_FRET_ (5′-FAM-[TAA-CCC]_4_-TAMRA-3′; donor fluorophore FAM is 6-carboxyfluorescein; acceptor fluorophore TAMRA is 6-carboxytetramethylrhodamine) was prepared as a 400 nM solution in 10 mM sodium cacodylate buffer at the respective pH and thermally annealed. Strip-tubes (QIAgen) were prepared by aliquoting 10 μl of the annealed DNA, followed by addition of Cu^2+^ solution and 2× sodium ascorbate solution to give the desired Cu^+^ concentration range across the samples, and made up with 10 mM sodium cacodylate buffer to a final volume of 20 μl. Fluorescence melting curves were acquired in a QIAgen Rotor-Gene Q-series polymerase chain reaction machine. Measurements were made with excitation at 483 nm and detection at 533 nm. Experiments were performed at each pH in triplicate with final analysis of the data carried out using QIAgen Rotor-Gene Q-series software and Origin.

### ^1^H NMR

^1^H nuclear magnetic resonance (NMR) experiments were performed using a Bruker Avance III 800 MHz spectrometer equipped with an HCN inverse triple resonance z-gradient probe. Aqueous solutions were prepared with the addition of 5% D_2_O to enable field/frequency lock. Solvent suppression of the water resonance was achieved using a 1D Watergate sequence employing a symmetrical 3-τ-9-τ-19 pulse train inversion element. The solvent resonance, which was minimized, was set on-resonance at the transmitter offset and the interpulse delay time (τ) was adjusted to achieve an excitation maximum in the imino proton region of interest. hTeloC was diluted to a concentration of 10 μM in 50 mM sodium cacodylate buffer at pH 5.5 containing 5% D_2_O. The spectrum of hTeloC alone was measured over 1 h after which 150 μM of Cu^+^ was added and the subsequent spectrum acquired over 2 h. At last, excess DETC (540 μM) was added and the spectrum acquired again for 1 h. NMR spectra were acquired and processed using Bruker’s TopSpin™ software package (v3.1.7 Bruker Biospin) for NMR data analysis.

### Computational methods

The starting point for our i-motif structural model was the PDB entry: 1EL2 (21), which was manually edited (22) to match the hTeloC sequence. This model, stabilized by six C^+^–C base pairs, was relaxed using a 200 ns explicit solvent molecular dynamics run to allow minor conformational changes in response to the altered sequence. The force field consisted of the OL15 parameters for DNA (23–25), TIP3P (26) model for water and Li, Song and Merz parameters for the ions (27). Partial charges for the protonated C^+^ bases were obtained by the RESP fitting procedure (see Supplementary Data). After equilibration, the 200 ns molecular dynamics simulation was performed at constant pressure and temperature (NPT, *P* = 0.1 MPa, T = 300 K) by using the Gromacs package (28).

The model of the C–Cu^+^–C i-motif was created from the final snapshot of the molecular dynamics run. Six Cu^+^ ions were inserted between matching cytosine groups, replacing the H^+^ ions, and three in the TAA loop regions. Optimization of this initial model with the force field described above gave an unexpected result with the Cu^+^ ions moving out of the planes of the cytosine rings. To validate this finding, we turned to the semi-empirical method PM6-D3H4 (29,30), which we had found to give good approximations to DFT results on isolated C–Cu^+^–C complexes (Supplementary Table S1). Optimization of the initial model with the Cu^+^ ions inserted by the PM6-D3H4 method gave the final structural model. Unfortunately, DFT-based optimization of the complete i-motif structure was not computationally feasible. As another approximation, a stack of six C–Cu^+^–C base pairs and the neighboring T and A residues were extracted from the initial model and optimized by the DFT method TPSS-D3(BJ)/def2-SV(P). Both the semi-empirical and DFT models featured the non-planar C–Cu^+^–C linkages.

Interaction energies between Cu^+^ or Cu^2+^ and the surrounding DNA residues (Supplementary Tables S1 and 2) were estimated both by using planar C–Cu–C complexes and by taking a fragment from the above DFT optimized stack, which consisted of two Cu ions and five bases (with additional geometry optimization to locate an energy minimum for Cu^2+^). The interaction energies were calculated by dispersion corrected DFT methods with def2-TZVP basis and include counterpoise correction for the basis set superposition error. All DFT calculations were performed by using the NWChem package (31).

## RESULTS AND DISCUSSION

**Figure 2.**
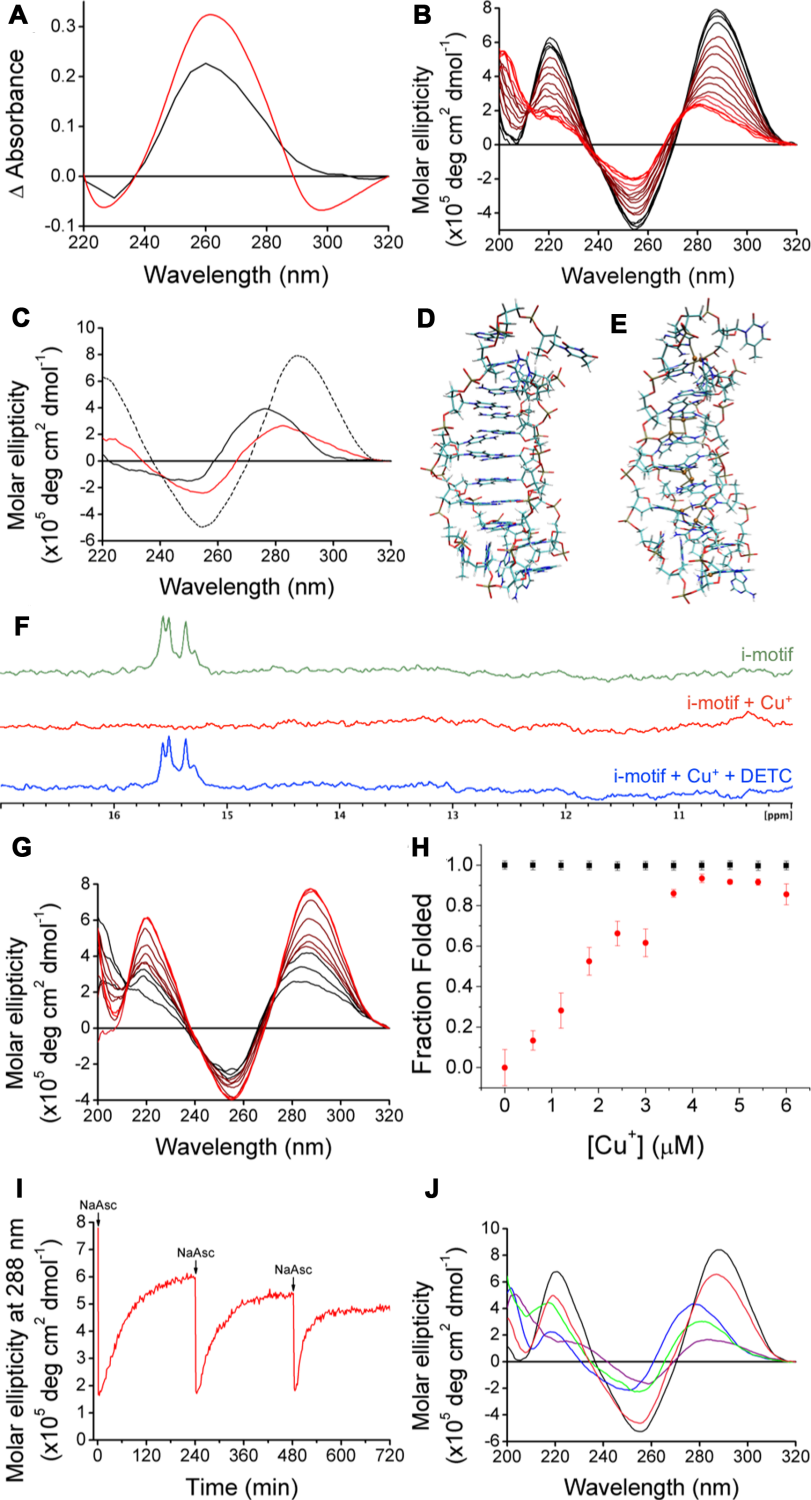
(**A**) ‘Cu^+^-difference’ spectra using 125 μM of Cu^+^ to form the final conformations at pH 7.4 (red) and pH 5.5 (black). (**B**) CD spectra of 10 μM hTeloC at pH 5.5 (black) with titration up to 150 μM Cu^+^ (red). (**C**) CD spectra of 10 μM hTeloC at pH 5.5 (dashed black), after addition of 150 μM Cu^+^ (red) or 1 mM Cu^2+^ (black). (**D**) Model of i-motif structure stabilized by protonation of C residues, snapshot from the end of the 200 ns simulation. (**E**) Model of the i-motif structure stabilized by Cu^+^ ions, derived from (D) by geometry optimization with the PM6-D3H4 method. (**F**) ^1^H NMR of (green) 10 μM hTeloC in 50 mM sodium cacodylate buffer pH 5.5 with 5% D_2_O; (red) addition of 150 μM Cu^+^; (blue) addition of 150 μM Cu^+^ and 540 μM DETC. (**G**) CD spectra of 10 μM hTeloC with 150 μM Cu^+^ at pH 5.5 (black) with titration up to 300 μM DETC (red). (**H**) Fluorescence intensity at 25°C normalized using values in the absence of Cu^+^ at pH 5.5 as 1 (folded) and at pH 7.4 as 0 (unfolded). A total of 200 nM hTeloC_FRET_ in 10 mM buffer at pH 5.5 (black) and at pH 7.4 (red). Error bars show standard deviation across three repeats. (**I**) Change in molar ellipticity at 288 nm of 10 μM hTeloC at pH 5.5 with 150 μM Cu^2+^ as a function of time with three additions of 150 μM sodium ascorbate under ambient conditions. (**J**) CD spectra of single sample of 10 μM hTeloC at pH 5.5 (black); addition of 1 mM Cu^2+^ (blue); addition of 150 μM sodium ascorbate (purple); after 4 h exposure to air (green); chelation using 1 mM EDTA (pink).

To investigate if Cu^+^ can induce the formation of a secondary structure in DNA, we used the cytosine-rich human telomeric DNA sequence hTeloC 5′-[TAA-CCC]_4_-3′ that is predominantly unfolded at physiological pH, but capable of forming i-motif at acidic pH (pH < 6). Cu^+^ in solution is well known to oxidize readily to Cu^2+^ when exposed to O_2_ (32,33), therefore all experiments were performed at ambient temperature under strict anoxic conditions in an N_2_ atmosphere (containing < 0.5 ppm O_2_). The UV absorbance profile of DNA is dependent on its conformation. Therefore, UV spectroscopy can be used to elucidate whether DNA is folded or unfolded, and to reveal the existence of higher-order secondary structure(s) (34). UV-difference spectra are used to identify and characterize the behavior of the secondary structure in response to experimental conditions (35,36). ‘Cu^+^ difference’ spectra for hTeloC were measured at pH 5.5 and pH 7.4, where the structure is an i-motif or unfolded DNA, respectively. The resulting spectra (Figure 2A) display a positive signal at 260 nm at both pH values, and a negative signal at 295 nm at pH 7.4, both consistent with when the i-motif formed by decreasing the pH (37). These results indicate that the final configuration of the secondary structure adopted at either pH is similar, and that at pH 7.4 a more substantial reconfiguration is necessary to form the final structure.

To further characterize the structure adopted by hTeloC in the presence of Cu^+^ we employed CD spectroscopy. The CD spectrum of hTeloC at pH 7.4 has a positive peak at 270 nm and a negative peak at 250 nm, indicative of a primarily unfolded population of oligonucleotide (38). Sequential addition of Cu^+^ up to five equivalents (i.e. 50 μM final) resulted in a bathochromic shift in the positive peak from 270 to 278 nm, while the position of the negative peak at 250 nm remained constant (Supplementary Figure S1). However, further addition of Cu^+^ at this pH resulted in visible precipitation of the Cu^+^–DNA complex and consequent deterioration of the CD signal. At pH 5.5, hTeloC is already folded into an i-motif with a characteristic positive peak at 288 nm and negative peak at 255 nm (38). Under these conditions, titration of Cu^+^ up to 19.5 equivalents (195 μM Cu^+^) led to a hypsochromic shift of the positive peak from 288 to 283 nm, and a decrease in the amplitude of the negative peak at 255 nm (Figure 2B). In contrast to the precipitation observed at pH 7.4, the Cu^+^–DNA complex at acidic pH was completely soluble beyond the concentration where no further changes are observed (150 μM Cu^+^). The changes observed at pH 7.4 and 5.5 are consistent with a Cu^+^ induced reconfiguration of the structure. Crucially, post-Cu^+^ addition and at both pH values, the spectra are practically superimposable indicating that a similar final structure is adopted regardless of pH.

Given the spectroscopic changes previously observed with Cu^2+^ and hTeloC, the possibility that the structure adopted in the presence of Cu^+^ may also display hairpin-like character (19) was explored, and the different copper–DNA complexes were compared using CD at pH 5.5. At this pH, in the absence of copper the CD spectrum of hTeloC has a positive peak at 288 nm indicative of i-motif structure. Addition of either Cu^+^ or Cu^2+^ resulted in a hypsochromic-shift consistent with an alteration in the structure of the DNA. Addition of Cu^2+^ shifts this peak to 276 nm compared to only 283 nm when Cu^+^ is added. The negative peak at 255 nm also undergoes a hypsochromic-shift to 250 nm in the presence of Cu^2+^, while the peak position does not shift at all when Cu^+^ is added (Figure 2C). This strongly suggests that the Cu^2+^–DNA complex is different to the Cu^+^–DNA complex.

In addition to the spectroscopic differences observed using the different oxidation states of copper, the half-cation concentrations also vary by an order of magnitude. A value of 46 (±3) μM was determined for the [Cu^+^]_50_, while the [Cu^2+^]_50_ was comparatively higher at 382 (±14) μM (Supplementary Figure S2). We previously suggested that the relatively high concentration of Cu^2+^ required to form the hairpin was because the Cu^2+^ did not affect the structure by interacting directly with the bases in the oligonucleotide, but rather by shifting the equilibrium to the hairpin structure via stabilization of the sugar–phosphate backbone (19). In the case of Cu^+^, continuous variation binding analysis determined the stoichiometry of Cu^+^ to DNA to be 9:1 (Supplementary Figure S3). This ratio further suggests that Cu^+^ is interacting differently to Cu^2+^, possibly via direct mediation of an interaction between bases in the sequence, perhaps consistent with the model proposed by Oomens: one Cu^+^ for each C–C base pair and additionally, one for each loop.

To compare the viability of C–C base pairs stabilized by Cu^+^ and Cu^2+^, models similar to the one shown in Figure 1C were created with both ions and optimized by DFT computational methods (Supplementary Figure S8) (31). The results revealed a planar C–Cu^2+^–C complex similar to the model of Oomens, but with a symmetrical structure and both the N and O atoms from a single face of each cytosine moiety coordinating the metal ion. Notably, the interaction energy between the two bases and the cation is sensitive to the redox-state of the metal, and is significantly larger for Cu^2+^ (-1700 kJ/mol) than for Cu^+^ (-650 kJ/mol). However, the experimental hydration enthalpies of the ions suggest that hydration is more energetically favorable to base pair formation involving Cu^2+^ (-2100 kJ/mol), compared to Cu^+^ (-593 kJ/mol) (39); which are consistent with the different behavior observed experimentally with these cations.

The CD spectra of the DNA at both pH values in the presence of Cu^+^ lay somewhere between that of unfolded DNA and i-motif DNA. Addition of Cu^+^ at pH 5.5 resulted in what appears to be slight unfolding of the i-motif, consistent with the structure expanding to accommodate the Cu^+^ cations, which are significantly larger than the protons which were previously stabilizing the C^+^–C base pairs. The potential folded i-motif structures were investigated in more detail using molecular modeling. First, a model of protonated hTeloC was created on the basis of the reported NMR structure from a similar sequence (Figure 2D) (21). The manually modified structure was optimized and then relaxed in a 200 ns explicit solvent molecular dynamics simulation (Supplementary Figure S9). Cu^+^ ions were added to this relaxed model manually (22). Six Cu^+^ ions were placed at the geometric midpoints between the N3 atoms of matching cytosine groups and three additional ions were placed in the TAA loop regions. The geometry of this initial Cu^+^–DNA complex structure was optimized using the semi-empirical PM6-D3H4 method (Figure 2E and Supplementary Figure S10) (29,30). In the optimized structure the Cu^+^ ions showed a preference to interact with more than two bases, thereby breaking the planarity of the C–Cu^+^–C units. Nevertheless, the overall folded structure was retained. To confirm these observations, a stack of six C–Cu^+^–C base pairs capped at both ends with the nearest molecular fragments was extracted from the initial Cu^+^–DNA complex and optimized using a DFT [TPSS-D3(BJ)/def2-SV(P)] method (Supplementary Figure S11). Both the semi-empirical and DFT calculations confirmed the preference of Cu^+^ ions to interact with more than two bases. Full exploration of the folding with Cu^+^ would require derivation and fitting of specific Cu^+^ force field parameters, which is beyond the scope of this work. Nevertheless, the computational modeling indicates the acid-stabilized and copper-stabilized i-motif structures are slightly different, which would explain the spectroscopic differences observed between these two species. We suggest the planar base pairing (C–Cu^+^–C) model may only be true for cytosine monomers. When the cytosines form part of a larger secondary structure, the interactions are more complex which gives rise to a slightly different i-motif structure, as supported by our spectroscopic data.

We have previously shown that the effects of Cu^2+^ on the structure of hTeloC DNA can be reversed using a chelator (19). To determine if a similar reversibility can be achieved with Cu^+^, the high-affinity chelator DETC was used in this work. Titration of DETC into hTeloC at pH 5.5 reverses the effects of the Cu^+^ addition and the structure reverts to that of the acid-stabilized i-motif. hTeloC with Cu^+^ had the positive peak at 283 nm, incremental titration of the chelator DETC resulted in a red-shift of the peak until it returned to the position of the acid-stabilized i-motif peak at 288 nm (Figure 2G). The negative peak in the presence of Cu^+^ at 255 nm did not shift its position but the amplitude of the signal increased to be more consistent with that of the original acid-stabilized i-motif.

Further experiments to examine the mode of copper binding to hTeloC were performed using ^1^H NMR. At pH 5.5, imino proton signals can be observed at 15.5 ppm and are characteristic of the C^+^–C base pairs in an acid-stabilized i-motif (40). On addition of Cu^+^, these signals disappeared, consistent with Cu^+^ replacing the protons in that position. This is in-line with the model proposed by Oomens and co-workers (20). Furthermore, no additional signals appeared, ruling out a hairpin conformation with additional Watson–Crick base pairing, as was seen with Cu^2+^ (19). The addition of the chelator DETC caused the NMR spectrum to return to that of the acid-stabilized i-motif, with chelation of Cu^+^ resulting in the reappearance of the imino proton signal at 15.5 ppm (Figure 2F).

Thus far, all experiments were carried out under stringent anoxic conditions to prevent the oxidation of Cu^+^ that would occur in the open air. We were interested in exploring whether redox-linked structural rearrangement would be observed *in situ* if the Cu^+^–DNA complex was exposed to air and the metal oxidized. The Cu^+^-i-motif can be formed by adding 150 μM of Cu^+^ and, as Cu^+^ is a high-affinity ligand compared with Cu^2+^, complete oxidation to Cu^2+^ yields a cation concentration below the [Cu^2+^]_50_ (382 ± 14 μM). As a result, there would not be enough Cu^2+^ to fully stabilize the hairpin. To test this hypothesis, Cu^+^ was added to hTeloC at pH 5.5 and the sample was split into two. One sample was maintained in an anoxic environment while the other was exposed to the open air. The CD spectrum for each condition was subsequently measured and the one which had been exposed to oxygen reverted almost completely to the acid-stabilized i-motif: the positive peak moved from 281 to 286 nm, and the amplitude of the negative peak at 255 nm increased, as observed when the Cu^+^ was chelated with DETC. In contrast, the sample maintained in the anoxic environment remained essentially unchanged (Supplementary Figure S4).

Having discovered that this system was oxygen-responsive and that oxidation of the Cu^+^ resulted in the restoration of the acid-stabilized i-motif structure we were interested in determining whether this transition was possible in the opposite direction; i.e. whether it would be possible to reduce Cu^2+^*in situ* to form the Cu^+^-i-motif. To explore this, the well-established reaction between Cu^2+^ and sodium ascorbate where Cu^2+^ is reduced to Cu^+^ was used (41). 150 μM Cu^2+^ was added to the acid-stabilized i-motif and, as this concentration is below the [Cu^2+^]_50_, no structural change was observed using CD. Subsequent addition of 150 μM sodium ascorbate resulted in the successful formation of the Cu^+^–i-motif complex observed previously when Cu^+^ was added under anoxic conditions (Supplementary Figure S5). When one equivalent of sodium ascorbate was used, exposure of the sample to air led to a slow process of oxidation and return to the acid-stabilized i-motif structure (Supplementary Figure S6), while adding an excess of sodium ascorbate allowed for the prolonged maintenance of the concentration of Cu^+^ and the corresponding Cu^+^ stabilized i-motif structure.

This ability to maintain the Cu^+^-i-motif structure for several hours in the open air allowed us to perform FRET-based DNA melting experiments using two equivalents of sodium ascorbate and observe the folding behavior using the dual-labeled sequence hTeloC_FRET_ 5′-FAM-[TAA-CCC]_4_-TAMRA-3′. The fluorescence signal at 25°C was used to determine the fraction of the DNA that is folded in the presence of increasing concentrations of Cu^+^. In good agreement with the UV and CD data, addition of Cu^+^ to hTeloC_FRET_ at pH 5.5 did not affect the proportion of the population of the DNA that was folded. Further ruling out unfolding and supporting the observations that the folded conformations of the proton-stabilized and Cu^+^-stabilized i-motif are similar. Conversely, at pH 7.4 addition of Cu^+^ results in folding of the sequence into a secondary structure that brings the two ends of the sequence into sufficient proximity for FRET to occur (Figure 2H). Additionally, using this technique we were able to determine a *T*_m_ which was calculated as the midpoint temperature of the transition from the folded to the unfolded structure. At pH 7.4 increasing Cu^+^ concentration led to an increase in *T*_m_ until 15 equivalents (3 μM) at which point it was 65°C, and after which no further change was observed. This is in agreement with the 15 equivalents of Cu^+^ required to fold the DNA as determined by CD. An increase in *T*_m_ was also observed at pH 5.5, however the temperature required to unfold completely is above 95°C, the limit of the instrument, therefore an accurate determination of the *T*_m_ was not possible (Supplementary Figure S7).

Having previously shown that multiple iterations of the conformational change by repeated chelation and metalation were possible using Cu^2+^ (19), we wanted to determine whether similar repeat switching was possible between the Cu^+^-stabilized and the acid-stabilized i-motif structures. From a nanotechnology perspective, the potential ability to have a conformational change of the structure controlled by redox-cycling the metal was very encouraging. To truly test the versatility of this system, rather than perform repeat additions of Cu^+^, we decided to perform a single addition of Cu^2+^ and reduce this *in situ* repeatedly to Cu^+^ using sodium ascorbate. The structural reconfiguration in response to the oxidation state of the copper was observed by monitoring the molar ellipticity at 288 nm as a function of time. Figure 2I shows the results of this experiment with three successive additions of the reducing agent successfully resulting in adoption of the Cu^+^-stabilized i-motif structure, and oxidation to Cu^2+^ over time similarly resulting in the return to the acid-stabilized i-motif prior to the next sodium ascorbate addition.

**Figure 3.**
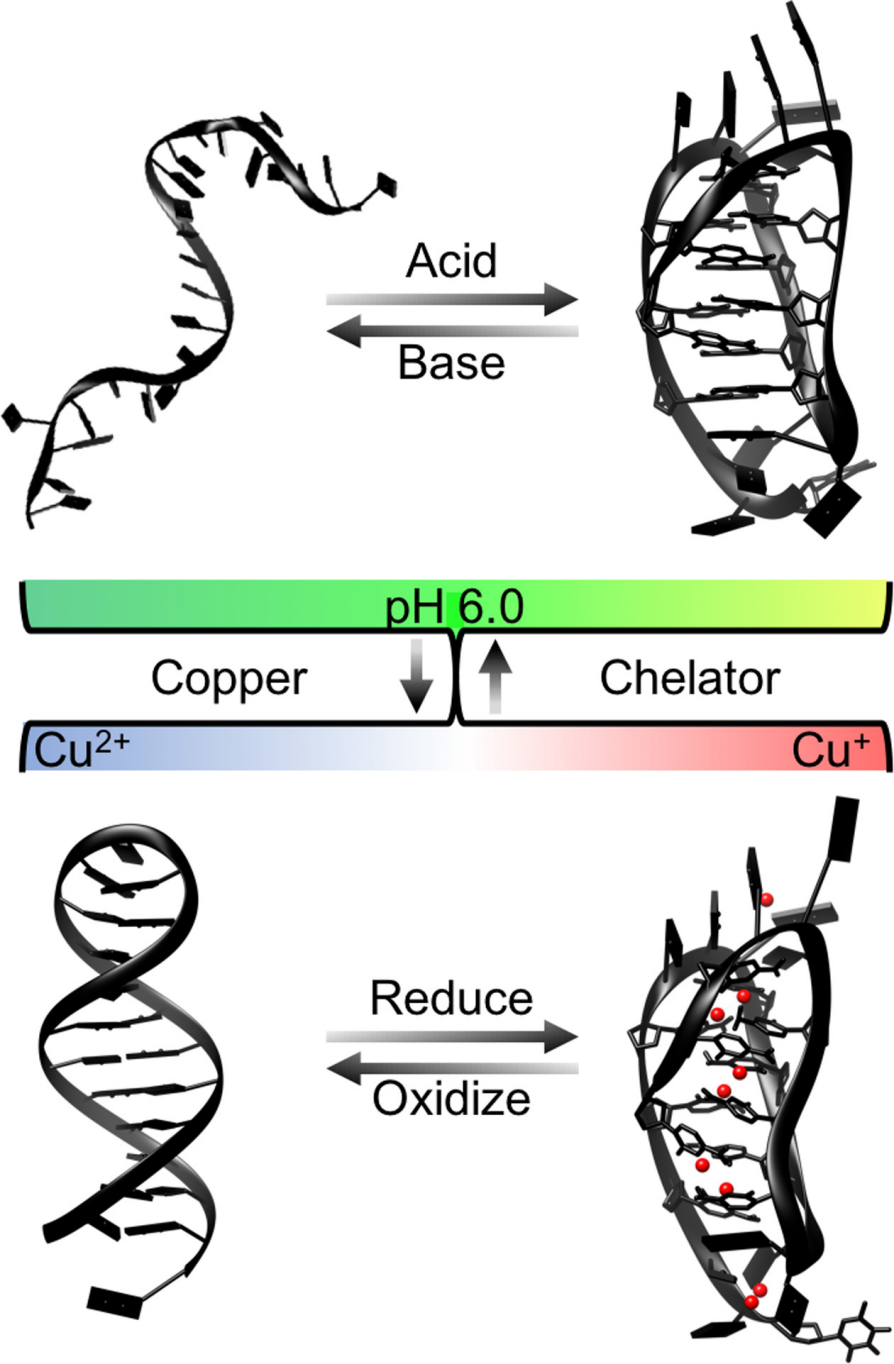
Illustration of proposed system for the pH and copper-redox-dependent control of the structure of the i-motif forming DNA sequence hTeloC.

Having established the redox-dependent coordination of copper by the i-motif forming DNA sequence hTeloC, we hypothesized that this system could act as a continuous redox-sensitive cycle, allowing for dynamic movement between the various structural conformations adopted under the different conditions. The final step was to determine whether it was possible to convert the Cu^2+^ hairpin structure to the Cu^+^-stabilized i-motif structure. As can be seen in Figure 2J, addition of 1 mM Cu^2+^ to a sample of hTeloC at pH 5.5 forms the hairpin structure and subsequent reduction to Cu^+^ using 150 μM sodium ascorbate successfully forms the previously observed Cu^+^–DNA i-motif, even in the presence of excess Cu^2+^; which is predictable due to the difference of an order of magnitude between the binding affinities of the different oxidation states of copper. Leaving the same sample in the open air over time resulted in conversion back to the Cu^2+^ stabilized hairpin structure. At last, addition of 1 mM ethylenediaminetetraacetic acid (EDTA) chelated the Cu^2+^ and the sample returned to its initial configuration as an acid-stabilized i-motif (Figure 2J). A summary of the transitions possible is conveyed in Figure 3, illustrating the proposed pH and redox sensitive control of the structural conformation of the i-motif forming DNA sequence hTeloC in the presence of copper.

This research demonstrates that Cu^+^ can be used to fold an i-motif forming DNA sequence into a Cu^+^-stabilized i-motif structure. This process can be reversed by chelation of the metal, or by oxidation of Cu^+^ to Cu^2+^. To our knowledge, this is the first example of redox-sensitive control of DNA secondary structure. This work realizes that a series of alternative conformational switches for i-motif forming DNA sequences are possible using different conditions, without changing the pH. The dynamics of this system could be applied to develop dual oxygen and pH-sensitive nanomachines, logic gates or sensors based on i-motif DNA.

## Supplementary Material

Supplementary Data
