# Supplementary material for: Redox-dependent control of i-Motif DNA structure using copper cations
Source: Nucleic Acids Res. 2018 May 24;46(12):5886–93. doi: 10.1093/nar/gky390 (PMC6159522; doi:10.1093/nar/gky390)
Supplement: Supplementary Data [file gky390_supplemental_files.docx]

**Redox dependent control of i‑motif DNA structure using copper cations**

Mahmoud A. S. Abdelhamid,^1,2^ László Fábián,^1^ Colin J. MacDonald,^1^ Myles R. Cheesman^2,3^ Andrew J. Gates^2,4^ and Zoë A. E. Waller^1,2*^

^1^ School of Pharmacy, University of East Anglia, Norwich Research Park, Norwich, NR4 7TJ, UK

^2^ Centre for Molecular and Structural Biochemistry, University of East Anglia, Norwich Research Park, Norwich, NR4 7TJ, UK

^3^ School of Chemistry, University of East Anglia, Norwich Research Park, Norwich, NR4 7TJ, UK

^4^ School of Biological Sciences, University of East Anglia, Norwich Research Park, Norwich, NR4 7TJ, UK


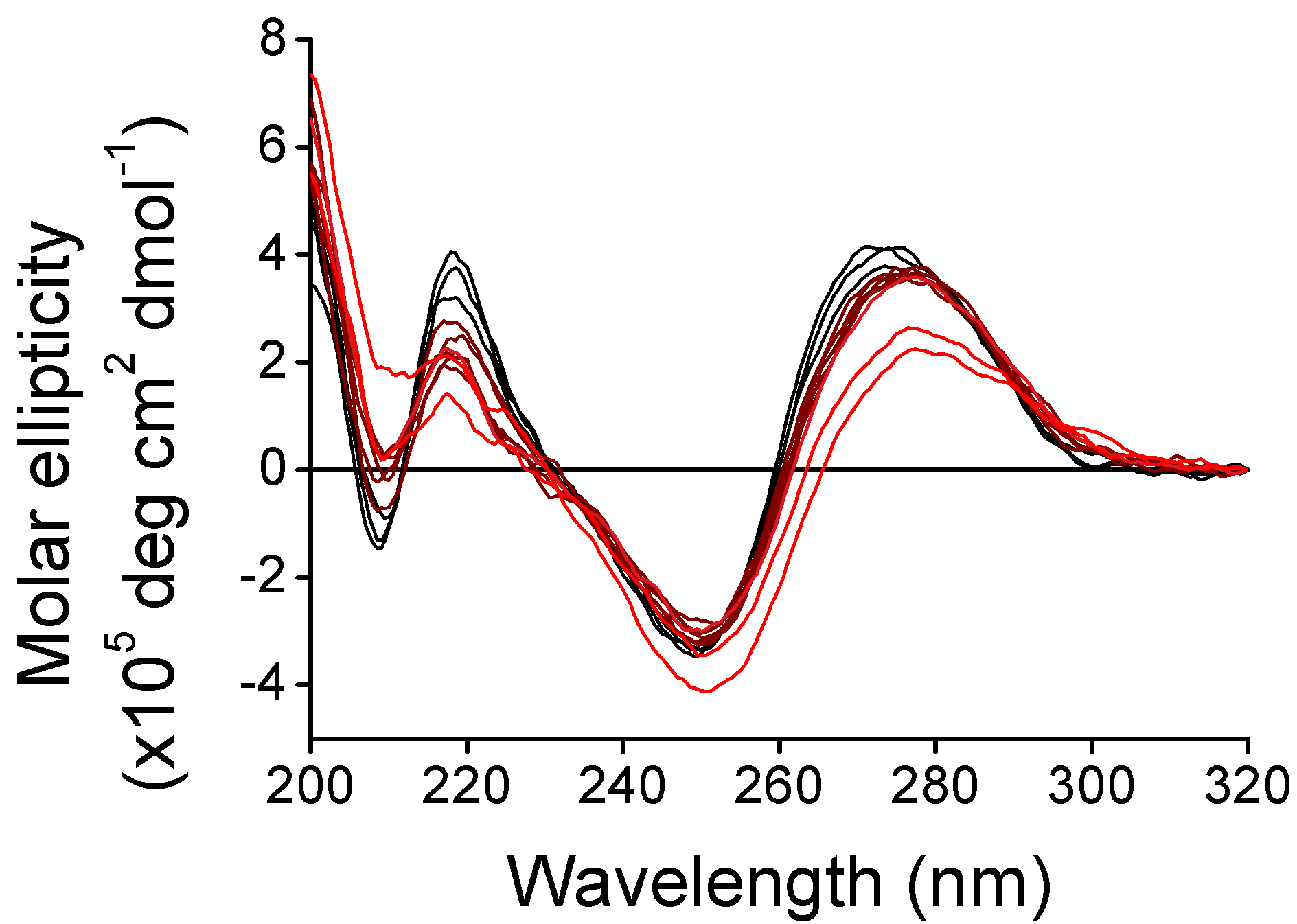


**Figure S1.** CD spectra of 10 µM hTeloC in 50 mM sodium cacodylate buffer at pH 7.4 with titration up to 50 µM Cu^+^. Experiment performed under anoxic conditions.


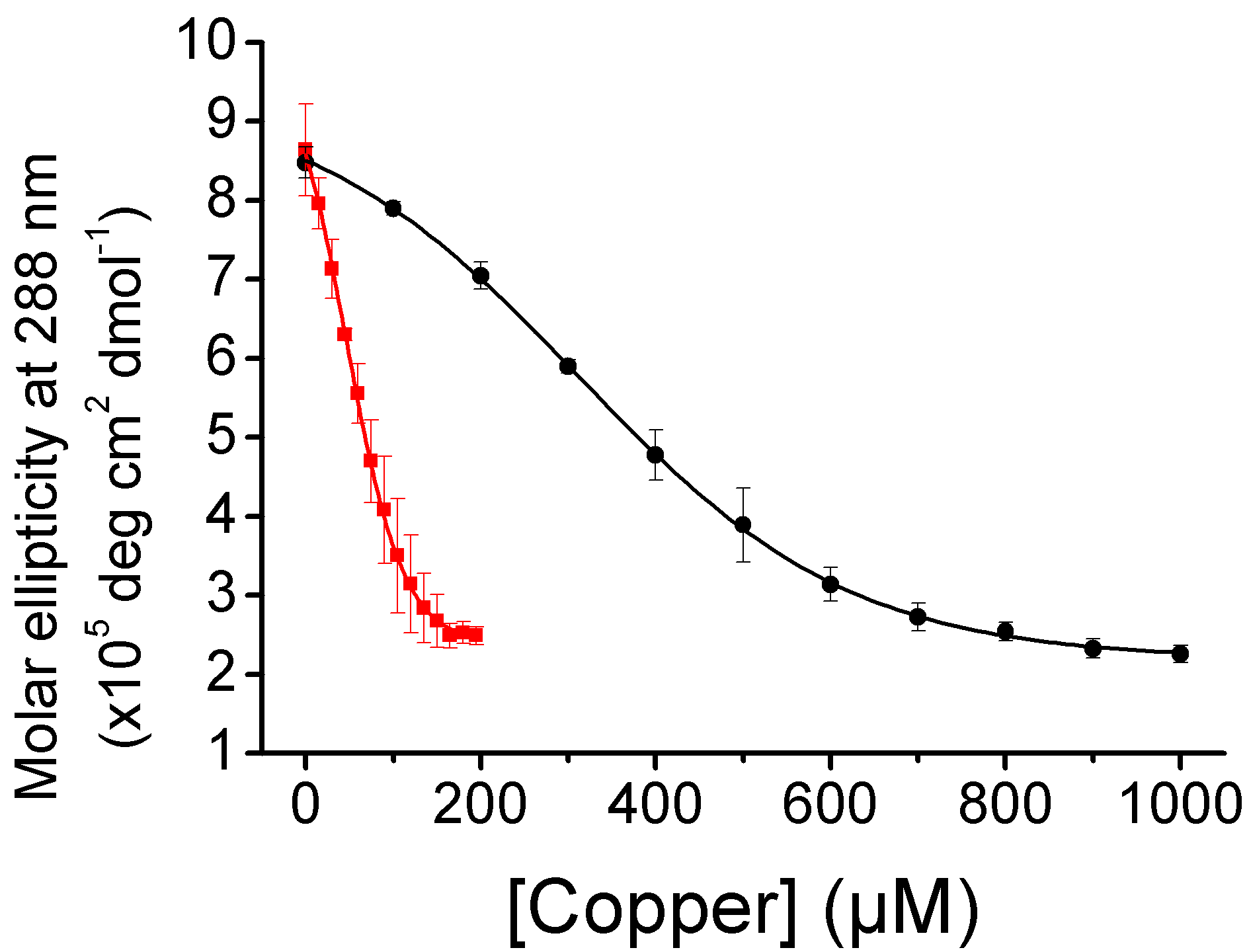


**Figure S2.** Change in molar ellipticity at 288 nm of 10 µM hTeloC in 50 mM sodium cacodylate buffer at pH 5.5 with titration up to 195 µM Cu^+^ (red) and of 10 µM hTeloC in 10 mM sodium cacodylate buffer at pH 5.5 with titration up to 1 mM Cu^2+^ (black). Error bars show standard deviation across three repeats. Cu^+^ experiments performed under anoxic conditions.


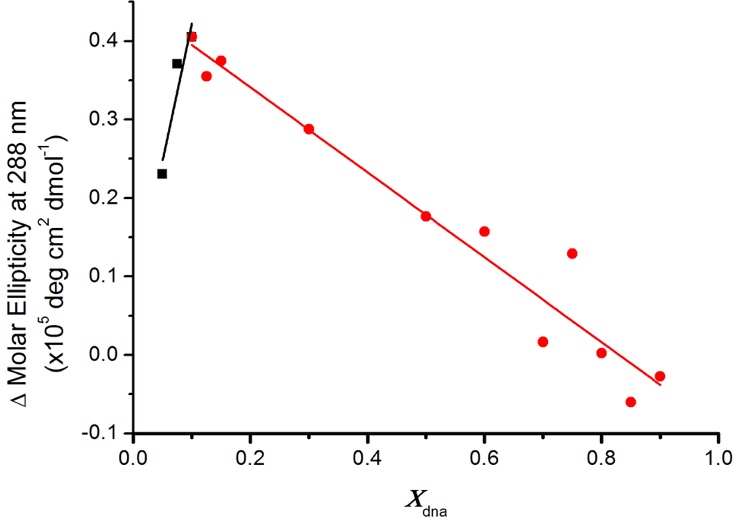


**Figure S3.** Job plot of hTeloC and Cu^+^ in 50 mM sodium cacodylate buffer at pH 5.5. The black and red symbols represent the points used for fitting the respective linear best fits to determine the intercept.


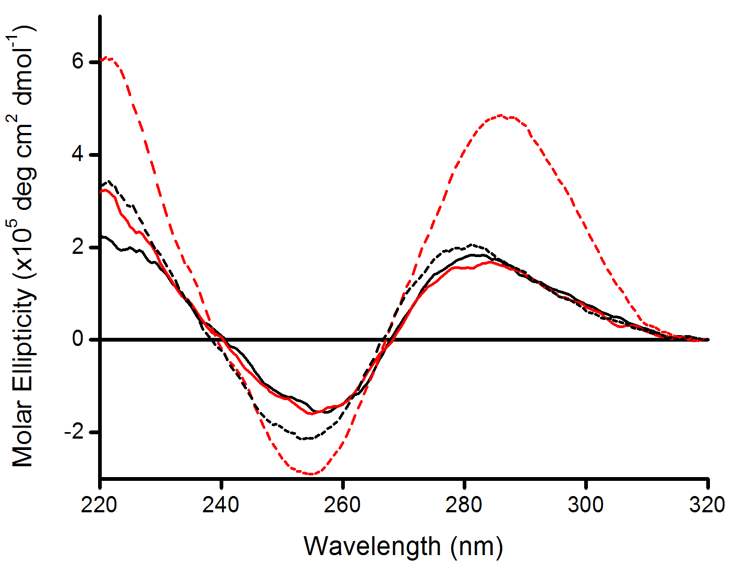


**Figure S4.** CD spectra of 10 µM hTeloC in 50 mM sodium cacodylate buffer at pH 5.5 with 150 µM Cu^+^ scanned immediately after addition (solid lines) and 3 hours later (dashed lines); (red) sample exposed to the air; (black) sample maintained in anoxic environment.


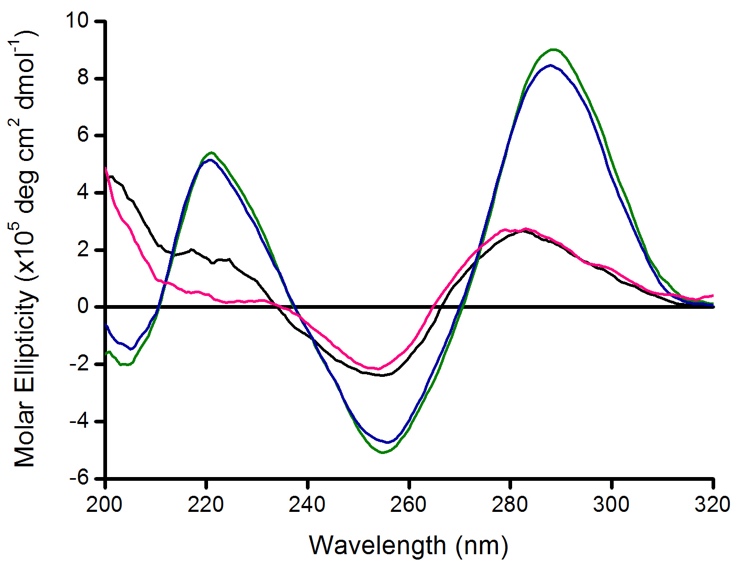


**Figure S5.** CD spectra of 10 µM hTeloC in 50 mM sodium cacodylate buffer at pH 5.5 (green); addition of 150 µM Cu^2+^ (blue); addition of 150 µM sodium ascorbate (pink); for comparison, the CD spectra of 10 µM hTeloC in 50 mM sodium cacodylate buffer at pH 5.5 after addition of 150 µM Cu^+^ (black). Slight discrepancy in signal intensity at wavelengths below 240 nm due to a corresponding absorbance observed in control sample of Cu^2+^ with sodium ascorbate in the absence of DNA at these wavelengths (data not shown).


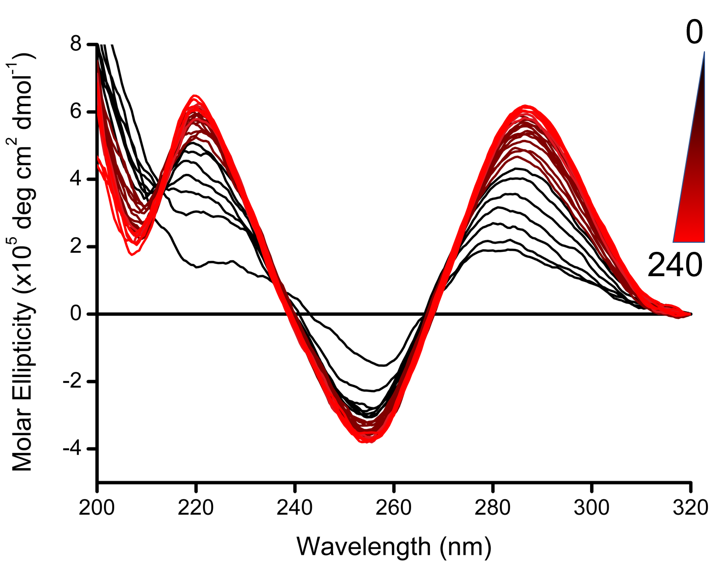


**Figure S6.** Example CD spectra of 10 µM hTeloC in 50 mM sodium cacodylate buffer at pH 5.5 with 150 µM Cu^2+^ immediately after addition of 150 µM sodium ascorbate and every 10 minutes for 240 minutes.

A
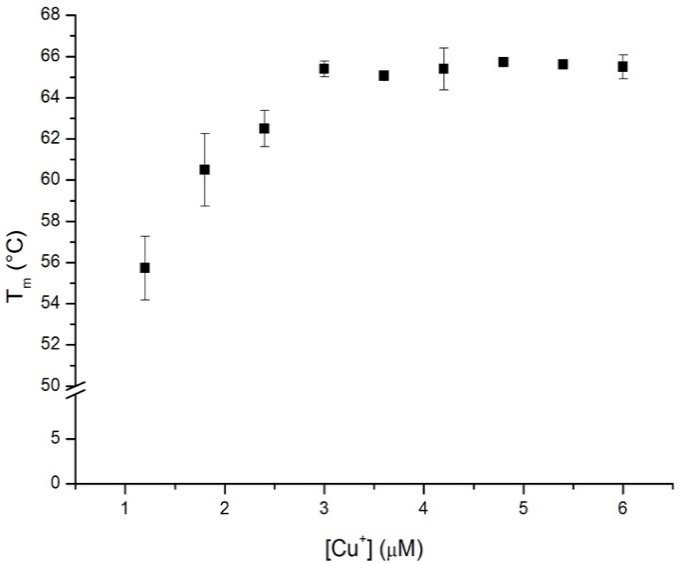


B
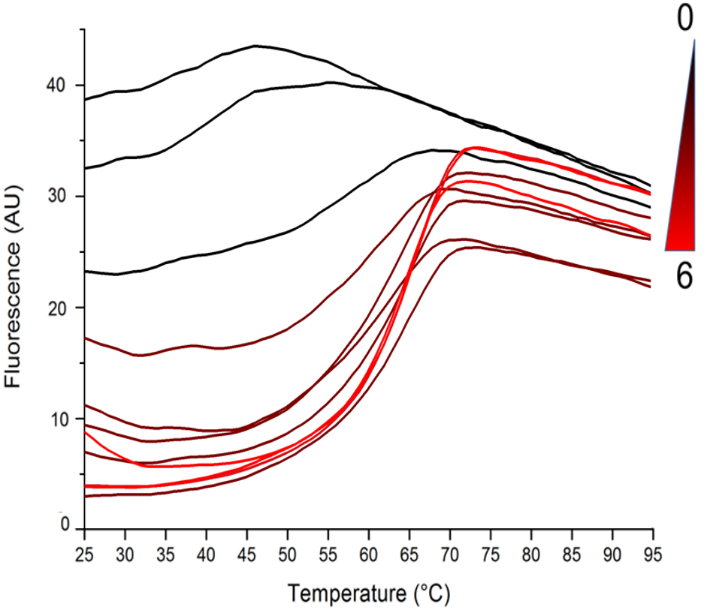


C
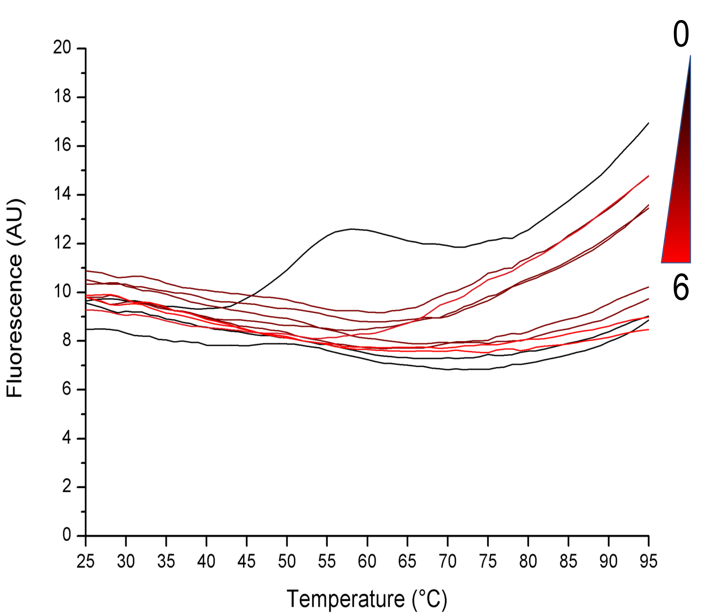


**Figure S7.** (A) *T*_m_ of 200 nM hTeloC in 10 mM sodium cacodylate buffer at pH 7.4 with 1.2 to 6 µM Cu^2+^ and 2 equivalents of sodium ascorbate. Error bars show standard deviation across three repeats. (B) Example FRET melting data of 200 nM hTeloC in 10 mM sodium cacodylate buffer at pH 7.4 with up to 6 µM Cu^2+^ and 2 equivalents of sodium ascorbate. (C) Example FRET melting data of 200 nM hTeloC in 10 mM sodium cacodylate buffer at pH 5.5 with up to 6 µM Cu^2+^ and 2 equivalents of sodium ascorbate.

**Optimisation of copper-stabilized cytosine base pairs**

Initial models of C-Cu^+^-C and C-Cu^2+^-C were constructed using the program Avogadro.^1^ These were optimized by the DFT methods B3LYP-D3/def2-TZVP and TPSS-D3(BJ)/def2-TZVP using the NWChem package.^2^ Both methods gave similar results (Figure S9, Table S1), with the Cu^+^-complex showing the same general features as reported by Oomens et al.^3^ The Cu^2+^-complex shows different metal ion coordination, with both the aromatic N and carbonyl O atoms of both bases at similar distances from the metal centre. Meanwhile, the NH…O hydrogen bonds are almost completely broken in the Cu^2+^-complex.

The semi-empirical PM6-D3H4 method,^4^ as implemented in Mopac2016,^5^ was also tested for the Cu^+^ complex. In comparison to the DFT methods, PM6-D3H4 favours stronger coordination to the carbonyl O atom at the expense of less ideal hydrogen bonding geometry (Table S1).

| 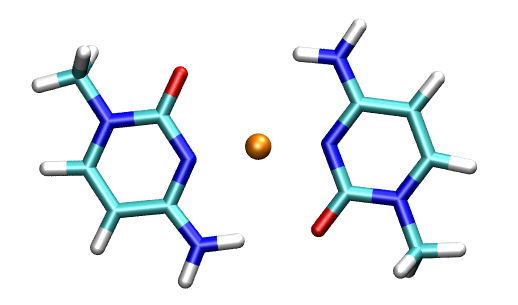 | 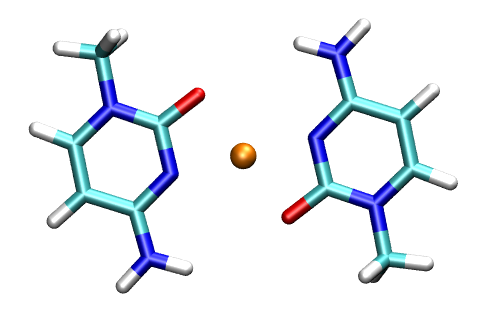 |
| --- | --- |
| (a) | (b) |
|  |  |

**Figure S8.** Structures of the (a) C-Cu^+^-C and (b) C-Cu^2+^-C copper-stabilized base pairs, determined by DFT [TPSS-D3(BJ)/def2-TZVP] geometry optimisation. Colour code: C: cyan, H: white, N: blue, O: red, Cu: gold.

**Table S1.** Structural and energetic details of the C-Cu^+^-C and C-Cu^2+^-C complexes

| **Metal ion** | **Cu^+^** | | | **Cu^2+^** | |
| --- | --- | --- | --- | --- | --- |
| **Computational method** | B3LYP-D3 / def2-TVZP | TPSS-D3(BJ) / def2-TZVP | PM6-D3H2 | B3LYP-D3 / def2-TZVP | TPSS-D3(BJ) / def2-TZVP |
| **Structure** | | | | | |
| **Cu-N (Å)** | 1.91, 1.91 | 1.88, 1.88 | 1.98, 1.98 | 2.00, 2.00 | 1.98, 1.98 |
| **Cu-O (Å)** | 2.74, 2.84 | 2.73, 2.84 | 2.14, 2.16 | 2.01, 2.01 | 2.02, 2.02 |
| **(N)H...O (Å)** | 2.24, 3.40 | 2.20, 3.25 | 2.60, 3.56 | 3.11, 3.11 | 3.08, 3.08 |
| **∠[N-H...O] (°)** | 176, 163 | 179, 165 | 146, 140 | 138, 138 | 138, 138 |
| **Energy** | | | | | |
| **Interaction energy (kJ/mol)** | -643.7 | -683.4 | -641.0 | -1675.8 | -1738.0 |
| **BSSE (kJ/mol)** | 7.5 | 7.8 | N/A | 8.9 | 8.3 |
| **BSSE corrected interaction energy (kJ/mol)** | -636.2 | -675.6 | N/A | -1666.8 | -1729.7 |

The interaction energies between the three components were calculated as

E(interaction) = E(C-Cu-C) – E(Cu) – 2 E(C),

where E(C-Cu-C) and E(C) are the total energies of the complex and the separate cytosine base at their respective optimum geometries and E(Cu) is the energy of an isolated copper (Cu^+^ or Cu^2+^) ion. The basis set superposition error (BSSE) was estimated by the counterpoise method at the optimum geometry of C-Cu^+^-C and C-Cu^2+^-C, respectively.

**Modelling the protonated hTeloC i-motif structure**

The starting point for the model was the 1EL2 PDB entry,^6^ a solution NMR based structure, with a highly similar sequence to hTeloC (CCCTAA5mCCCTAACCCUAACCCT). The 1EL2 model was manually edited in Avogadro^1^ to match the hTeloC sequence (TAACCCTAACCCTAACCCTAACCC). A molecular dynamics run was then performed to relax the modified structure, i.e., to allow minor conformational changes in response to the altered sequence.

The AmberTools 16 package^7^ was used to assign force field parameters to the model and to add 17 charge-compensating sodium ions. For DNA the OL15 parameter set was used,^8-10^ for the ions the parameters of Li, Song and Merz^11^ together with the TIP3P water model.^12^ The protonated cytosines were modelled using parameters taken from analogous atoms in neutral residues, with the exception of partial charges. These were fitted to reproduce the electrostatic potential of a geometry optimized B3LYP/6-31G* model of the protonated nucleoside. Only the charges of the protonated cytosine and the C1’-H1’ atoms of the sugar were taken from the RESP fitting procedure, where their sum was constrained to give a molecular charge of +1 when used in combination with standard force field charges for the rest of the sugar fragment.

After minimisation using an implicit solvent model with sander^7^ (part of AmberTools), the model was transferred to the Gromacs package^13^ by using a locally modified version of the amber_to_gmx_by_XL script.^14^ Calculated energies between the two packages agreed to within 0.1%. The model was solvated in a cubic box with a 63.37 Å axis (8057 water molecules). The positions of the water molecules and sodium ions were energy minimized and then equilibrated using constant volume and temperature (NVT, T = 300 K) molecular dynamics for 100 ps, followed by a constant pressure and temperature simulation (NPT, p = 0.1 MPa) for 200 ps. In subsequent equilibration runs (1 ns NVT, 2 ns NPT) the coordinates of the DNA as well as the solvent molecules and counterions were allowed to change. The length of the production run was 200 ns. Each simulation used a time step of 2 fs.

The basic structure of the i-motif was unchanged throughout the simulation, with the bases of each C^+^-C pair remaining in close proximity (Figure S10). The TAA fragments, which form loops, showed a much higher degree of flexibility.

| 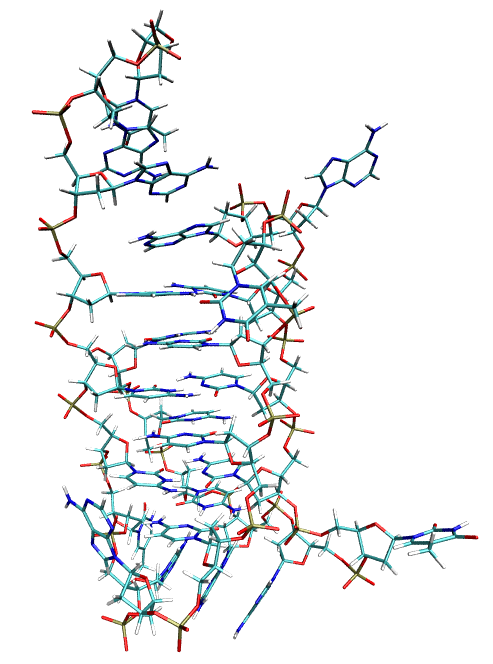 | 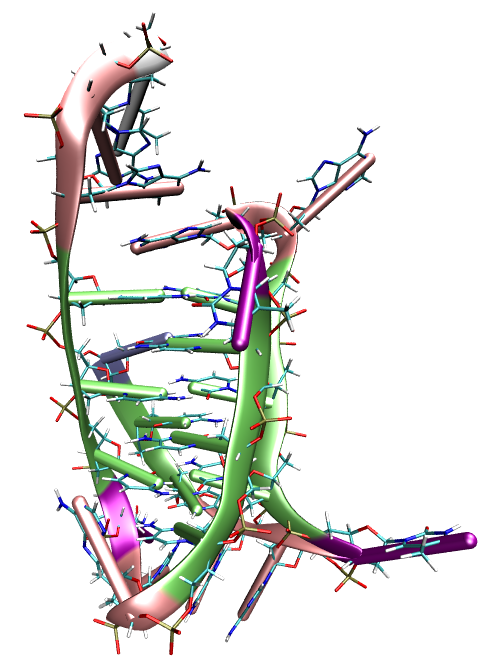 |
| --- | --- |
| (a) | (b) |
|  |  |
| 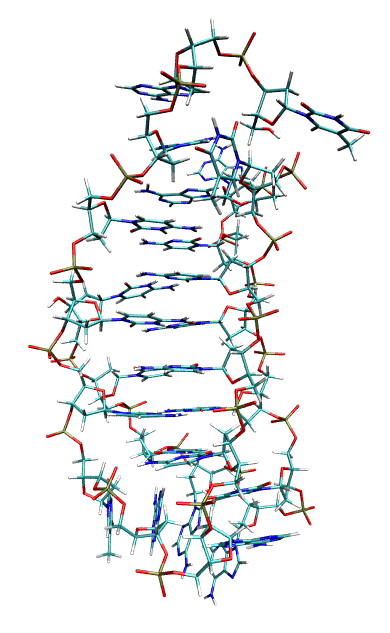 | 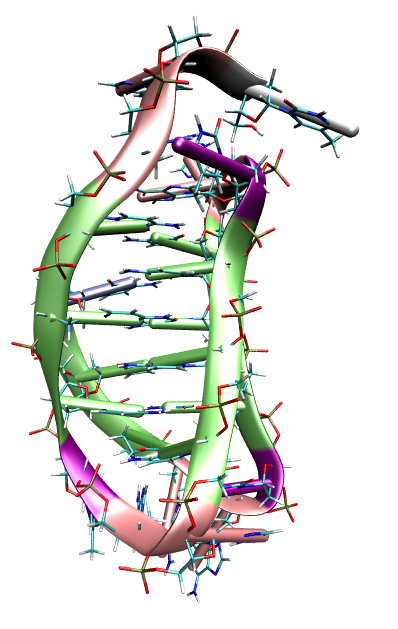 |
| (c) | (d) |
|  |  |

**Figure S9.** Representations of i-motif structures stabilized by protonation of C residues. The structure shown in (a) and (b) is a snapshot from the molecular dynamics simulation after 100 ns represented as (a) bonds and (b) tertiary structure cartoon. The model in (c) and (d) is a snapshot from the end of the 200 ns simulation. The colour code for the cartoons in (b) and (d) is 5’-T: white, 3’-C: grey, T: purple, A: pink, C: green.

**Modelling the Cu^+^ stabilized i-motif structure**

Models of the C-Cu^+^-C i-motif were created from the final, 200 ns snapshot of the protonation-stabilized structure (Figure S10). After deprotonation of the C^+^ residues, six Cu^+^ ions were inserted at the geometric midpoints between the N3 atoms of matching cytosine groups. To match the experimentally observed 9:1 Cu^+^ to DNA stoichiometry, three additional ions were placed in the TAA loop regions. Their initial positions were selected so as to allow coordination of each Cu^+^ with at least one aromatic N atom and ensuring they were surrounded by heteroatoms as much as possible, while avoiding unphysically short contacts. Optimisation of this initial model with the force field described above and the Li, Song & Merz HFE parameters^11^ for Cu^+^ gave an unexpected result. Instead of forming planar C-Cu^+^-C links, the Cu^+^ ions moved out of the planes of the cytosine rings and so were able to interact with more than two bases. The reliability of this observation is, of course, severely limited by the fact that the Cu^+^ parameters had been derived to describe the hydration of these ions, not their interaction with DNA.

Optimisation of the same initial structure with the semi-empirical method PM6-D3H4^4-5^ gave a qualitatively similar result (Figure S11). The orientations of the cytosine groups changed to allow Cu^+^ ions to interact with more than two bases, while the overall folding remained similar (see Figs. S10d and S11b).

| 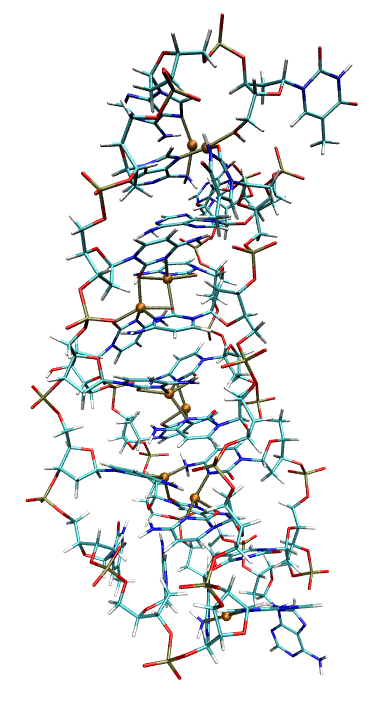 | 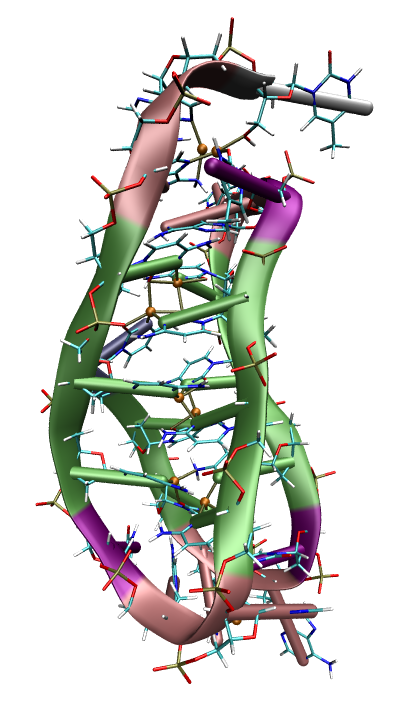 |
| --- | --- |
| (a) | (b) |
|  |  |

**Figure S10.** Representations of the i-motif structure stabilized by Cu^+^ ions. The model was obtained by geometry optimisation with the PM6-D3H4 method. The model was derived from the one shown in Figure S10(c, d).

Unfortunately, DFT-based optimisation of the complete i-motif model was not computationally feasible. In order to test the observed structural changes by a non-empirical method, the stack of six C-Cu^+^-C base pairs and the neighbouring T and A residues were extracted from the initial model. Each nucleobase was capped by a methyl group on the N1 atom. The resulting model was optimized by the DFT method TPSS-D3(BJ)/def2-SV(P). The geometry of the model in Figure S12a is not fully converged (max. gradient = 9 x 10^-4^ a.u., rms gradient = 2 x 10^-4^ a.u.), because there is a weak repulsion between the top two and bottom four ions and their ligands. On full optimization, these parts drift away from each other, giving the structures in Figure S12b and S12c, respectively. Of course, in the i-motif structure the separation of the two parts would be prevented by the DNA backbone.

Despite the model representing only a truncated part of the i-motif structure, the DFT results clearly support the previous findings and show structural rearrangements that allow Cu^+^ ions to have threefold coordination. This is in contrast to the direct analogy with C^+^-C and expectations based on the simple C-Cu^+^-C model calculations, but could be the reason for the observed differences between the CD spectra of the protonation and the Cu^+^ stabilized folded structures.

The interaction energy between the metal ions and the bases was estimated by using the geometry optimised assembly shown in Figure S12b and the B3LYP-D3(BJ)/def2-TZVP level of DFT theory (Table S2). The total interaction energy, E(interaction), was broken down into a ‘coordination’ component, E(coord), which gives the interaction between the metal ions and the bases and the intermolecular interaction energy between the bases, E(intermol):

E(coord) = E(assembly) – 2 E(Cu) – E(bases),

E(intermol) = E(bases) – E(A) – 4 E(C),

E(interaction) = E(coord) + E(intermol) = E(assembly) – 2 E(Cu) – E(A) – 4 E(C),

where E(assembly) is the total energy of the assembly shown in Figure 12b, E(bases) is the energy of the same assembly with the ions removed, E(C) and E(A) are the total energies of the separate cytosine and adenine bases, respectively and E(Cu) is the energy of an isolated copper ion. The basis set superposition error (BSSE) was estimated by the counterpoise method. For comparison, the same assembly was optimised after replacing Cu^+^ by Cu^2+^ and the resulting geometry was used to estimate the interaction energy in a hypothetical Cu^2+^ analogue. Similarly to the planar models discussed earlier (Table S1), the results suggest that hydration is more favourable (-2100 kJ/mol) for Cu^2+^ than the formation of such a complex. For Cu^+^, on the other hand, the energy of complex formation is more favourable than hydration (-593 kJ/mol).

| 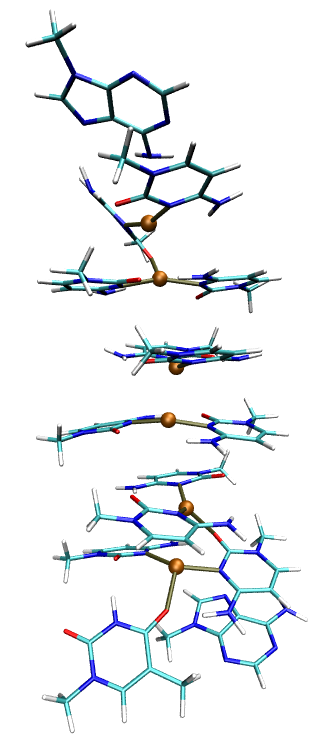 | 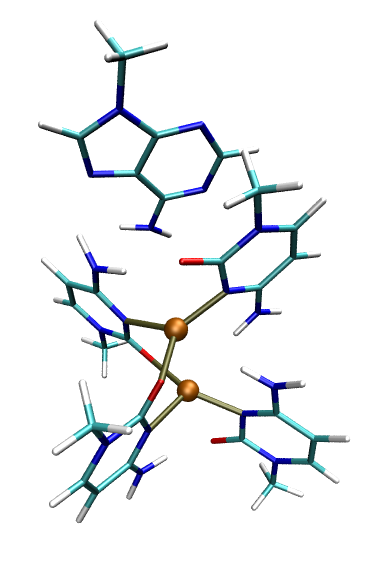 |
| --- | --- |
|  | (b) |
|  |  |
|  | 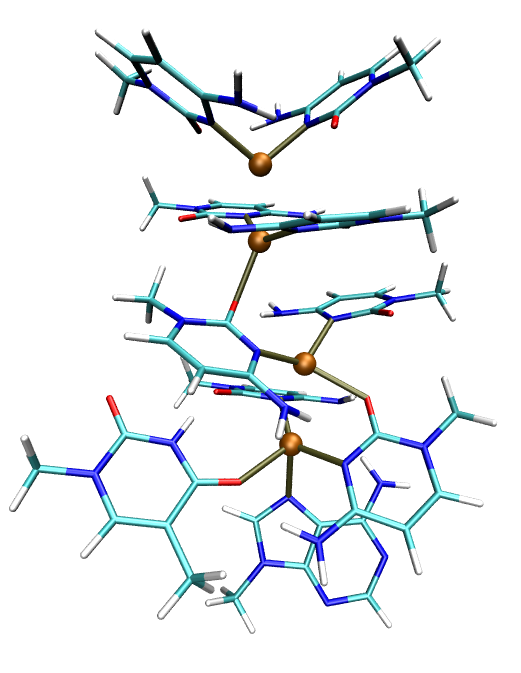 |
| (a) | (c) |
|  |  |

**Figure S11.** DFT optimized structures of a stack of C-Cu^+^-C pairs extracted from the i-motif model, showing (a) a loosely converged full model and (b,c) fully converged parts of the model that drifted apart during optimisation.

**Table S2.** Interaction energies calculated after geometry optimisation of the complex shown in Figure S12b using the B3LYP-D3(BJ)/def2-TVZP // TPSS-D3(BJ) / def2-SV(P) level of theory. All terms are corrected for BSSE.

| **Metal ion** | **Cu^+^** | | **Cu^2+^** | |
| --- | --- | --- | --- | --- |
|  | Total interaction | Interaction / ion | Total interaction | Interaction / ion |
| **Interaction between the metal ions and bases, E(coord) (kJ/mol)** | -1227.3 | -613.6 | -2760.0 | -1380.0 |
| **Interaction between the five bases, E(intermol) (kJ/mol)** | -7.9 | -4.0 | 239.0 | 119.5 |
| **Total energy of interaction, E(interaction) (kJ/mol)** | -1235.2 | -617.6 | -2521.0 | -1260.5 |

SI References:

1. Hanwell, M. D.; Curtis, D. E.; Lonie, D. C.; Vandermeersch, T.; Zurek, E.; Hutchison, G. R., Avogadro: an advanced semantic chemical editor, visualization, and analysis platform. *J Cheminform* **2012,** *4* (1), 17.

2. Valiev, M.; Bylaska, E. J.; Govind, N.; Kowalski, K.; Straatsma, T. P.; Van Dam, H. J. J.; Wang, D.; Nieplocha, J.; Apra, E.; Windus, T. L.; de Jong, W. A., NWChem: A comprehensive and scalable open-source solution for large scale molecular simulations. *Computer Physics Communications* **2010,** *181* (9), 1477-1489.

3. Gao, J.; Berden, G.; Rodgers, M. T.; Oomens, J., Interaction of Cu + with cytosine and formation of i-motif-like C–M + –C complexes: alkali versus coinage metals. *Phys. Chem. Chem. Phys.* **2016,** *18* (10), 7269-7277.

4. Řezáč, J.; Hobza, P., Advanced Corrections of Hydrogen Bonding and Dispersion for Semiempirical Quantum Mechanical Methods. *Journal of Chemical Theory and Computation* **2012,** *8* (1), 141-151.

5. Stewart, J. J. P., MOPAC 2016. Stewart Computational Chemistry, Colorado Springs, CO, USA: 2016.

6. Phan, A. T.; Guéron, M.; Leroy, J.-L., The solution structure and internal motions of a fragment of the cytidine-rich strand of the human telomere 1 1Edited by I. Tinoco. *Journal of Molecular Biology* **2000,** *299* (1), 123-144.

7. Case, D. A.; Cerutti, D. S.; Iii, T. E. C.; Darden, T. A.; Duke, R. E.; Giese, T. J.; Gohlke, H.; Goetz, A. W.; Greene, D.; Homeyer, N.; Izadi, S.; Kovalenko, A.; Lee, T. S.; LeGrand, S.; Li, P.; Lin, C.; Liu, J.; Luchko, T.; Luo, R.; Mermelstein, D.; Merz, K. M.; Monard, G.; Nguyen, H.; Omelyan, I.; Onufriev, A.; Pan, F.; Qi, R.; Roe, D. R.; Roitberg, A.; Sagui, C.; Simmerling, C. L.; Botello-Smith, W. M.; Swails, J.; Walker, R. C.; Wang, J.; Wolf, R. M.; Wu, X.; Xiao, L.; York, D. M.; Kollman, P. A., AMBER 2017. University of California, San Francisco: 2017.

8. Krepl, M.; Zgarbová, M.; Stadlbauer, P.; Otyepka, M.; Banáš, P.; Koča, J.; Cheatham, T. E.; Jurečka, P.; Šponer, J., Reference Simulations of Noncanonical Nucleic Acids with Different χ Variants of the AMBER Force Field: Quadruplex DNA, Quadruplex RNA, and Z-DNA. *Journal of Chemical Theory and Computation* **2012,** *8* (7), 2506-2520.

9. Zgarbová, M.; Luque, F. J.; Šponer, J.; Cheatham, T. E.; Otyepka, M.; Jurečka, P., Toward Improved Description of DNA Backbone: Revisiting Epsilon and Zeta Torsion Force Field Parameters. *Journal of Chemical Theory and Computation* **2013,** *9* (5), 2339-2354.

10. Zgarbová, M.; Šponer, J.; Otyepka, M.; Cheatham, T. E.; Galindo-Murillo, R.; Jurečka, P., Refinement of the Sugar–Phosphate Backbone Torsion Beta for AMBER Force Fields Improves the Description of Z- and B-DNA. *Journal of Chemical Theory and Computation* **2015,** *11* (12), 5723-5736.

11. Li, P.; Song, L. F.; Merz, K. M., Systematic Parameterization of Monovalent Ions Employing the Nonbonded Model. *Journal of Chemical Theory and Computation* **2015,** *11* (4), 1645-1657.

12. Jorgensen, W. L.; Chandrasekhar, J.; Madura, J. D.; Impey, R. W.; Klein, M. L., Comparison of simple potential functions for simulating liquid water. *The Journal of Chemical Physics* **1983,** *79* (2), 926-935.

13. Abraham, M. J.; Murtola, T.; Schulz, R.; Páll, S.; Smith, J. C.; Hess, B.; Lindah, E., Gromacs: High performance molecular simulations through multi-level parallelism from laptops to supercomputers. *SoftwareX* **2015,** *1-2*, 19-25.

14. Li, X. amber_to_gmx_by_XL. <https://github.com/recoli/amber_to_gmx_by_XL> (accessed 04/08/17).
